# Bacterial and fungal communities in tracheal aspirates of intubated COVID-19 patients: a pilot study

**DOI:** 10.1038/s41598-022-13482-w

**Published:** 2022-06-14

**Authors:** Alicia Ruiz-Rodriguez, Paula Lusarreta-Parga, Wouter A. A. de Steenhuijsen Piters, Lilian Koppensteiner, Carlos E. Balcazar-Lopez, Robyn Campbell, Rebecca Dewar, Martin P. McHugh, David Dockrell, Kate E. Templeton, Debby Bogaert

**Affiliations:** 1 Centre for Inflammation Research, University of Edinburgh, 47 Little France Crescent, Edinburgh, EH16 4TJ UK; 2 Department of Paediatric Immunology and Infectious Diseases, Wilhelmina Children’s Hospital/University Medical Center Utrecht, Lundlaan 6, 3584 EA Utrecht, The Netherlands; 3 Department of Laboratory Medicine, Royal Infirmary of Edinburgh, 51 Little France Crescent, Edinburgh, EH16 4SA UK; 4 School of Medicine, University of St Andrews, North Haugh, St Andrews, KY16 9TF UK

**Keywords:** Infectious-disease diagnostics, Bacterial infection, Fungal infection, Viral infection

## Abstract

Co-infections with bacterial or fungal pathogens could be associated with severity and outcome of disease in COVID-19 patients. We, therefore, used a 16S and ITS-based sequencing approach to assess the biomass and composition of the bacterial and fungal communities in endotracheal aspirates of intubated COVID-19 patients. Our method combines information on bacterial and fungal biomass with community profiling, anticipating the likelihood of a co-infection is higher with (1) a high bacterial and/or fungal biomass combined with (2) predominance of potentially pathogenic microorganisms. We tested our methods on 42 samples from 30 patients. We observed a clear association between microbial outgrowth (high biomass) and predominance of individual microbial species. Outgrowth of pathogens was in line with the selective pressure of antibiotics received by the patient. We conclude that our approach may help to monitor the presence and predominance of pathogens and therefore the likelihood of co-infections in ventilated patients, which ultimately, may help to guide treatment.

## Introduction

SARS-CoV-2 (severe acute respiratory syndrome Coronavirus 2) is a respiratory virus that causes coronavirus disease 19 (COVID-19). It emerged in December 2019 and has caused a global pandemic. As a consequence, SARS-CoV-2, has been responsible for high numbers of hospitalizations and deaths worldwide. Clinical manifestations of COVID-19 strongly differ between individuals, ranging from asymptomatic and mild infections to severe disease. Risk factors for severe disease are obesity, older age and pre-existing comorbidities such as hypertension, diabetes and chronic lung disease^1^. Severe COVID-19 disease has shown to be driven by aggressive inflammatory responses as a result of a complex interplay of viral load, immune response and patient comorbidities^2^ with potentially other drivers involved, such as bacterial and fungal co-infections. On top of this, critically ill patients with COVID-19 are at high risk of ventilator-associated pneumonia caused by hospital-acquired multidrug-resistant organisms, which might prolong duration of mechanical ventilation and hospitalization^3^. Interestingly, during hospital-acquired pneumonia, the lung microbiome appears to be characterized by a relatively high biomass and overgrowth of one or more pathogens, having major consequences for the host response during and after pneumonia^4^.

Therefore, in the context of the highly variable course of COVID-19 infection, understanding dysbiosis of the respiratory microbiome might offer new insights into both disease severity and the role of co-infections during ventilation. To date, few studies have investigated the respiratory microbiome in COVID-19 patients. Overall, they confirmed predominance of multiple pathobionts and oral commensal bacteria in lower respiratory tract samples^5–9^, the latter supporting the idea of micro-aspiration of oral microbes as a source of lung colonization and infection. Some studies have already linked the composition of microbial communities with COVID-19 disease severity^8,10,11^. Most of these studies confirm similar patterns in COVID-19 patients compared to patients diagnosed with community-acquired pneumonia in the pre-pandemic era^6^. Importantly, only a few of them incorporated the analysis of the fungal community in their study approach^6,9^, despite the known high risk of fungal infections in ICU patients in general^12^. Surveillance of fungal pathogens is crucial in critically ill patients because (i) there are emerging multi-drug fungi and (ii) fungi have a strong potential to colonize the host and participate in polymicrobial infections with bacteria^13^.

Irrespective, we hypothesize that rapid evaluation of the microbial community composition including information on outgrowth of individual pathogens may help to identify dysbiosis of the respiratory microbiome thereby offering a basis to monitor co-infections, offering a more comprehensive evaluation of risk scores and thereby potentially improving surveillance and tailored treatment. In this pilot study, we, therefore, assessed the absolute abundance and composition of the bacterial and fungal communities in endotracheal aspirates of intubated COVID-19 patients using next-generation sequencing targeting both bacteria and fungi.

## Results

### Characteristics of the study population

We obtained respiratory specimens from 30 critically ill patients admitted to the ICU with a COVID-19 infection. Patient demographics and clinical characteristics are reported in Table 1. We received a total of 42 samples from 30 patients, of which 8 of these patients had multiple samples taken across 3 time points, as reported in Table 2. Patients included in this study were all admitted to the ICU for a severe COVID-19 infection and most required mechanical ventilation. The samples were collected very early in the pandemic, i.e. on or prior to April 2, 2020, therefore, no vaccines, no steroids and no antivirals were administered to these patients.

**Table 1:** Demographics and clinical characteristics of the study cohort.

Patients characteristics (n = 30)	
Mean age (SD), years	58 (11)
Gender, male (%)	24 (80)
Patients intubated (%)	25 (83.33)
Days between hospitalization and intubation, median (range)	1 (0–9)
Days of intubation, median (range)	12 (0–140)
Outcome of the disease	
Alive (%)	20 (66.67)
Death (%)*	10 (33.33)

SD = standard deviation; range is expressed as minimum value–maximum value.

*All patients died in ICU (< 35 days following onset of symptoms).

**Table 2:** Patients samples information.

Sample-level information (n = 42)	T1	T2	T3
Patients samples per time point	30	8	4
Sample type			
Tracheal aspirate (%)	26 (86.67)	8 (100)	4 (100)
Sputum (%)	3 (10)		
Bronchoalveolar lavage (%)	1 (3.33)		
Days after admission, median (range)	2 (0–13)	7 (2–12)	8 (5–16)
Abx at the time of sampling, yes (%)	14 (46.67)	6 (75)	4 (100)
Abx administered intravenously			
Coamoxiclav (%)	2 (6.67)	1 (12.5)	0
Coamoxiclav plus Clarithromycin (%)	7 (23.33)	0	0
Amoxicillin plus Clarithromycin (%)	2 (6.67)	0	0
Vancomycin plus Ciprofloxacin (%)	1 (3.33)	1 (12.5)	0
Piperacillin plus Tazobactam (%)	1 (3.33)	3 (37.5)	3 (75)
Other (%)	1* (3.33)	1** (12.5)	1^†^ (25)

T = time point, Abx = antibiotic. * Doxycyclin. ** Vancomycin. ^†^ Vancomycin_Metronidazole.

### Overall microbiome composition of samples (Microbial density)

First, we studied the 16S-rRNA and ITS1 DNA concentration in samples and controls (Supplementary Fig. 2). The bacterial density was as expected generally higher in samples compared to the negative controls (Supplementary Fig. 2), whereas more overlap was found between fungal biomass in samples versus controls. Next, we removed potential contaminating reads from the sample profiles using *decontam* package. After quality control, filtering and removal of potential contaminants, a total of 562,305 sequences remained available for further analysis for the bacterial dataset (mean ± SEM, 13,388.21 ± 1,19.48 reads per sample). For the fungal dataset, a total of 879,482 sequences remained available for further analysis (mean ± SEM, 20,940.05 ± 4423.45 reads per sample). Within the bacterial dataset we observed 174 operational taxonomic units (OTUs), mostly *Proteobacteria* (41.85% of reads), followed by *Actinobacteria, Firmicutes, Bacteroidetes* and *Fusobacteria* (23.81%, 21.39%, 9.85% and 1.75% of reads, respectively). The fungal dataset consisted of 213 OTUs, dominated by *Ascomycota* (71.67% of reads), followed by *Basidiomycota* (27.88%).

### Bacterial community composition

**Figure 1:**
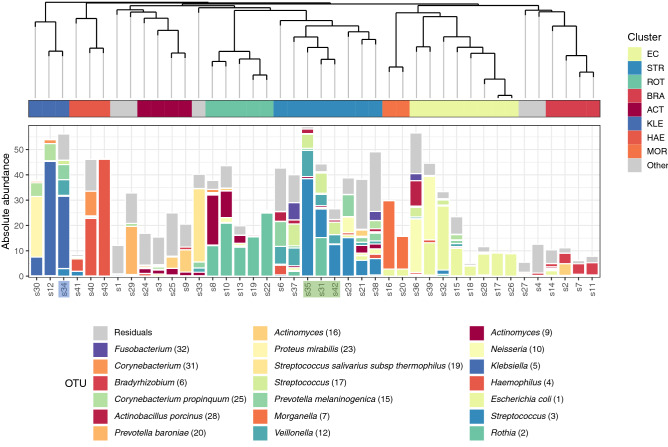
Respiratory bacterial communities in COVID19 patients. Dendrogram visualizing a hierarchical clustering of samples. Stacked bar charts show the absolute abundance of the 20 highest-ranked OTUs and of residual bacteria. On the basis of clustering indices, an optimal number of 13 clusters was identified, 8 of which comprised more than one study samples. Classifier taxa for these eight clusters were: *Escherichia coli* (EC), *Streptococcus* (STR), *Rothia* (ROT), *Bradyrhizobium* (BRA), *Actinomyces* (ACT), *Klebsiella* (KLE), *Haemophilus* (HAE) and *Morganella* (MOR). Gray mark individuals not included in any of these eight clusters. All the samples are tracheal aspirates except for s31, s35 and s42 that are sputum (highlighted in green) and s34 that is bronchoalveolar lavage (highlighted in blue).

To compare bacterial community structures across samples, we performed hierarchical clustering based on Bray–Curtis dissimilarities (Fig. 1). We identified eight clusters that contained more than one sample (n = 37). The bacterial biomass differed significantly between clusters (*p* < 0.05 Kruskal–Wallis rank sum test) (Supplementary Fig. 2), indicating that microbiota profiles were linked with bacterial biomass. Clusters dominated by *Streptococcus, Haemophilus* and *Klebsiella* species represented a high biomass. In contrast, mixed profiles were associated with a lower biomass.

The *Klebsiella*-dominated cluster (KLE-Cluster), representing three samples, showed the highest bacterial biomass. The high predominance of *Klebsiella* in combination with the high biomass may suggest bacterial co-infection in these patients. A similar combination between biomass and predominance was observed for the *Haemophilus*-dominated samples (HAE-Cluster). *Streptococcus-, Rothia-* and *Actinomyces-* dominated clusters (STR-Cluster, ROT-Cluster and ACT-Cluster, respectively) were typified by their respective predominating bacteria, in combination with the presence of a group of lower abundant ‘oral’ microbes. The STR-Cluster, despite showing a very high biomass, was very diverse, which might indicate either overgrowth of potentially pathogenic streptococci like *S. pneumoniae*, or recent aspiration. Interestingly, one sample within the STR-Cluster (sample 6) showed a relatively high abundance of *Mycoplasma* (36) (27.87% abundance). One of the major clusters was an *Escherichia coli*-dominated cluster (EC-cluster; 8 samples). Interestingly, though 6/8 samples within this cluster showed clear *E. coli* (1) predominance, two samples, both with a high biomass, were dominated by two bacterial OTUs, *E.coli* (2) and *Neisseria* (10). Also, the *Morganella*-dominated cluster (MOR-cluster) included two samples with high biomass and dual predominance of *Morganella* (7) and *E. coli* (1). The relatively high biomass clearly suggests bacterial out- or overgrowth. Finally, the *Bradyrhizobium-dominated* cluster (BRA-cluster) contained four samples, all with a low biomass. The fact that *Bradyrhizobium* is considered an environmental bacterium suggests a lower likelihood of a bacterial co-infection in these patients.

### Fungal community composition

**Figure 2:**
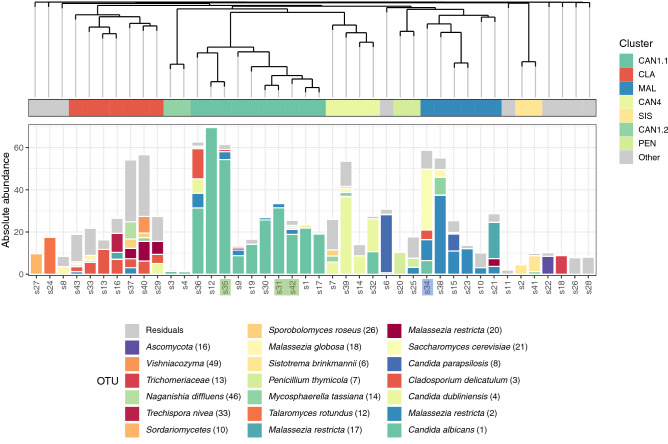
Respiratory fungal communities in COVID19 patients. Dendrogram visualizing a hierarchical clustering of samples. Stacked bar charts show the absolute abundance of the 20 highest-ranked OTUs and of residual bacteria. On the basis of clustering indices, an optimal number of 16 clusters was identified, 7 of which comprised more than one study samples. Classifier taxa for these seven clusters were: *Candida albicans* (CAN), *Cladosporium* (CLA), *Malassezia* (MAL), *Candida dubliniensis* (CAN4), *Sistotrema* (SIS) and *Penicillium* (PEN). Gray mark individuals not included in any of these eight clusters. *Candida albicans* (1) dominated two separate clusters, one comprised 10 samples, termed CAN1.1 and the second comprised two samples, termed CAN1.2-Cluster. Cluster dominated by *Candida dubliniensis* (4) is termed CAN4. All the samples are tracheal aspirates except for s31, s35 and s42 that are sputum (highlighted in green) and s34 that is bronchoalveolar lavage (highlighted in blue).

Next, we investigated the fungal community structure across all samples, again using a clustering approach (Fig. 2). We identified 7 clusters including more than one sample (n = 33). We observed large differences in fungal biomass between the clusters (*p* < 0.05 Kruskal–Wallis rank sum test) (supplementary Fig. 3). Clusters dominated by *Candida albicans* (CAN1.1-Cluster), *Cladosporium* (CLA-Cluster), *Candida dubliniensis* (CAN4-Cluster) and *Malassezia* (MAL-Cluster) showed a very high biomass compared to clusters dominated by *Sistrotema* (SIS-Cluster), *Penicillium* (PEN-Cluster) and a second small *C. albicans*-dominated cluster (CAN1.2-Cluster).

The major fungal cluster (CAN1.1-Cluster) was composed of 10 samples. Three of these samples showed a clear overgrowth of *C. albicans* (1) (> 50% of the absolute abundance), which was associated with high biomass, therefore suggestive of potential fungal co-infection. Though a second *C. albicans* cluster (CAN1.2-Cluster) showed high relative abundances of *C. albicans* (1), the biomass of these samples was very low. We interestingly observed a third *Candida* cluster, CAN4-Cluster, dominated by *C. dubliniensis* (4). The contribution of *C. dubliniensis* (4) to the total absolute abundance was more than 50% for 3 out of 4 samples, and interestingly, one of these samples was co-dominated by *C. dubliniensis* (4) and *C. albicans* (1).

The second largest cluster was the CLA-Cluster, including 7 samples. Though several of these samples had a high fungal biomass they were not clearly dominated by a single OTU. The fungal profiles of these samples were more evenly distributed, suggesting limited overgrowth of individual species. This is in line with co-presence of *Malassezia* in this cluster, suggesting merely a general commensal fungal presence or outgrowth rather than pathogenic predominance. The remaining clusters all showed a more diverse community. Combined with the low biomass, they are less suggestive of active fungal co-infections. Importantly, though *Aspergillus* taxa were observed in our patients (Supplementary Fig. 4), these had a relatively low absolute abundance, therefore not suggesting active *Aspergillus* infections.

### Associations between bacterial and fungal microbiota profiles

**Figure 3:**
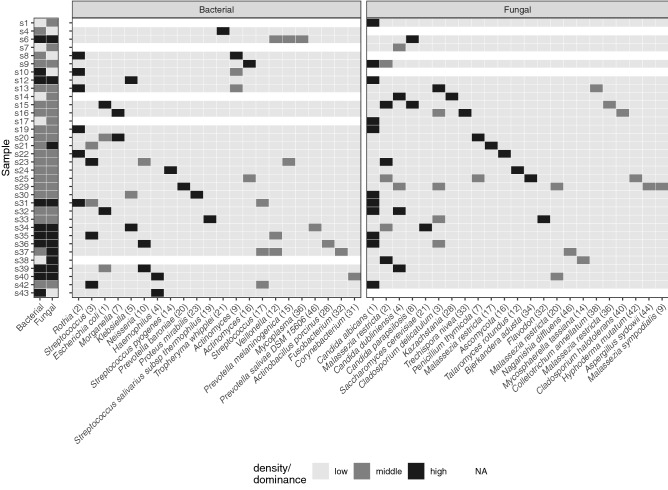
Heatmap depicting bacterial and fungal density and dominance of taxa on a per-sample basis. Only samples with a bacterial and/or fungal biomass higher than the 25th percentile are shown. Biomass data are shown by the first two columns; low (< 25th percentile), middle (25–75th percentile) or high (> 75th percentile). Relative abundance was stratified in low (< 15%), middle (15–30) and high (> 30%)-predominance. Only OTUs dominant (i.e. denoted ‘middle’ or ‘high’) at least in one sample were included (resulting in 23 bacterial OTUs and 25 fungal OTUs). Samples lacking any dominant OTU were not shown.

To explore potential cross-domain relationships, we assessed the association between bacterial and fungal biomass, and observed a positive significant linear relationship (Supplementary Fig. 5), indicating that patients with high bacterial biomass also presented with a high fungal biomass. Next, we investigated the potential association between fungal and bacterial taxa on a per-sample basis (Fig. 3). Interestingly, some samples showed clear overgrowth of bacterial and fungal pathogens, such as sample 12 that showed co-predominance with *Klebsiella* (5) and *C. albicans* (1) or sample 39 showing co-predominance of *E. coli* (1), *Neisseria* (10) and *C. dubliniensis* (4). These associations were further confirmed by Spearman's rank correlation analysis (Supplementary Fig. 6). In total, 52 bacterial-fungal pairs were identified, however, after correcting for multiple testing, only 7 remained. Of these, *Malassezia restricta* (2) was positively associated with members of the oral environment, such as *Streptococcus* (3 and 17), *Prevotellamelaninogenica* (15) and *Veillonella* (5). *Mycosphaereallatasianna* (14) was positively associated with *Haemophilus* (4) and *Fusobacterium* (32).

### Microbiota changes over time

**Figure 4:**
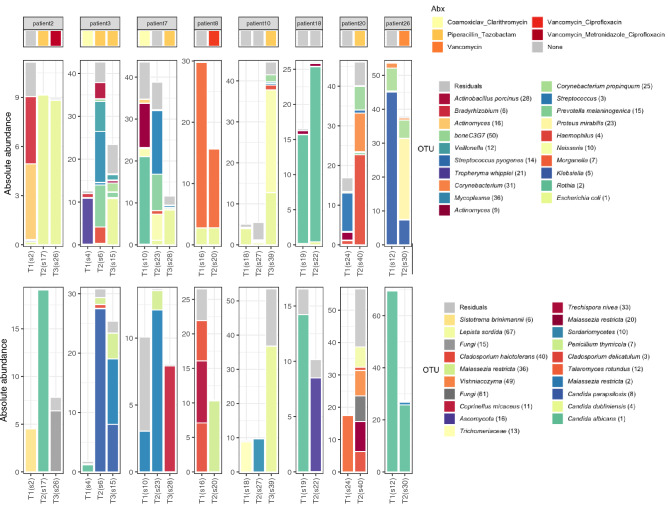
Microbiota changes over time on a per-patient basis. The first row displays antimicrobial treatment. Second and third rows show a stacked bar chart of the absolute abundance of the 20 highest-ranked OTUs and of residual bacteria and fungi respectively.

Follow-up samples were available for a limited number of patients (n = 8). Changes in their respiratory microbial community over time are shown in Fig. 4. Interestingly, we observed increases in abundance of bacteria such as *E. coli* over time in patients 2, 3, 7, and 10. In parallel, in patients 2, 3 and 10 we also found an increase in especially *Candida* abundance. Since all patients were treated with antimicrobials including antifungals in some, changes could be the result of antimicrobial pressure.

We further explored whether the initiation of antimicrobial treatment was related to changes in both biomass and community composition, we found for example in patient 10 that the introduction of antimicrobials (Piperacillin and Tazobactam) was related with consecutive bacterial and fungal outgrowth as well as an increase in abundance of *E. coli* (1), *Neisseria* (10) and *C. dubliniensis* (4). In patient 20, the introduction of Piperacillin and Tazobactam was followed by outgrowth of *Haemophilus* (4), *Corynebacterium* spp., and a very diverse fungal community, including *Aspergillus piperis* (80) (supplementary Fig. 4). In contrast, in patients 8 and 26, the introduction of systemic vancomycin or vancomycin plus ciprofloxacin, was related with a decrease in the bacterial and fungal biomass, though predominance of the respective pathogens remained.

Despite limited statistical power due to small sample size and an overall severe group of patients, we investigated the relation between microbial community composition and disease severity, measured as days of intubation and outcome of the disease using baseline samples only. Days since intubation did not differ significantly across bacterial and fungal clusters (*p* > 0.05 Kruskal–Wallis rank sum test, data not shown). Also, in this relatively small sample set, we found no association between bacterial and fungal clusters and outcome (*p* > 0.05 Chi-squared test, data not shown). A larger dataset is however needed to further explore relationships between microbial community changes and antibiotic treatment, as well as potential associations with patients’ outcomes.

## Discussion

We characterized the microbiota of lower respiratory tract samples of COVID-19 patients in ICU to describe potential microbial dysbiosis that could suggest potential bacterial and fungal co-infections. Most importantly, our methods combine information on bacterial and fungal biomass together with community profiling, resulting in a semiquantitative method using dual criteria for potential co-infections: (1) a high bacterial and/or fungal biomass combined with (2) predominance of individual potential pathogens. We observed a clear association between microbial outgrowth (high biomass) and predominance of individual species, suggesting the combination of data might help to determine the likelihood of co-infections and guide treatment in clinical practice.

The microbiota profiles we identified in our patients, showing predominance of common bacterial and fungal pathogens, are in sharp contrast to those observed in healthy lungs, where low biomass and a more diverse profile including mostly oral taxa, such as *Streptococcus*, *Veillonella*, *Prevotella*, *Neisseria* and *Porphyromonas* can be found^14^. Higher bacterial burden and/or the presence of gut-associated bacteria in BAL samples have previously been associated with acute respiratory distress syndrome (ARDS) and poor outcome in ICU patients^15,16^. Besides, previous evidence suggests that the lung microbiota is related to alveolar and systemic inflammation in critically ill patients^16^. Interestingly, we also observed besides well-known respiratory pathogens, enrichment with gut-associated pathogens, especially *Escherichia* spp., in our patients, which is in line with findings in patients with established ARDS^15^. Given the ARDS-like phenotype observed in a subset of COVID-19 patients^17^ and the critical stage of the patients included in our pilot study, one could speculate that lung microbiota may impact disease outcome of acute COVID-19, not only through infection, but also through immunomodulation. The association between resident microbiota and host responses was shown previously for both lower-^18^ and upper respiratory tract communities in the context of RSV in infants^19^ and in mild influenza infection in adults^20^. As such, microbiota-broad information might help to not only identify clear co- or superinfections, but also guide treatment on a personalized level, to reduce proinflammatory properties of lung microbiota, and thereby alter the course of disease in severely ill patients.

Recent studies confirm a low rate of bacterial and fungal co-infections in COVID-19 patients, though higher frequencies have been observed in ICU patients. So far, the main bacterial pathogens identified in hospitalized patients were *M. pneumonia*, *H. influenzae*, *P. aeruginosa*, *E. coli*, *Klebsiella* spp., and *S. aureus*^9,21–24^. Fungal pathogens so far identified in COVID-19 patients include *A. fumigatus* and *C. albicans*^21,24^. These results are in line with our study findings where *E. coli*, *Klebsiella*, *Haemophilus* and *C. albicans* were predominant pathogens within high biomass samples. Importantly, our protocol was able to detect the presence of *Aspergillus* in several samples. However, in this pilot study, we did not find evidence of *Aspergillus* predominance, despite other reports showing occasional aspergillosis in prolonged ventilated COVID-19 patients^21^. Compared to Fortarezza et al.^25^, our cohort is a younger population that did not receive corticosteroids and all samples were collected during the first wave of the pandemic, where the incidence of COVID-19 Associated Pulmonary Aspergillosis (CAPA) appeared lower, which might explain our findings.

We believe the described method could be a useful tool for monitoring potential overgrowth and pathogenic behaviour of bacteria and fungi in ventilated patients. This is valuable given fungal co-infection can be severe, though are often detected late or not at all^26^. Despite the proven low rate of bacterial/fungal co-infection in COVID-19, more than 70% of the patients receive antimicrobial therapy^22,27^. Under these conditions of antibiotics pressure, in combination with the inflammatory environment, resident or acquired fungi might be well able to overgrow^28^. In our pilot, we observed a clear expansion of the fungal community over time during antimicrobial treatment (piperacillin/tazobactam), whereas we found the opposite in samples from patients that received systemic vancomycin plus or minus ciprofloxacin or metronidazole. This indicated that fungal overgrowth might not only depend on antimicrobial pressure per se, but also on the antimicrobial class used and the microbial community structure they are part of. To date, however, only one small study from China has applied a microbiome approach to diagnosis of bacterial/fungal co-infections, and on upper instead of lower respiratory samples of COVID-19 patient^7^. They found that most of the upper respiratory samples from severe patients were mostly dominated by a single pathogen, (relative abundance > 60%) including *Burkholderia*, *Staphylococcus,* or *Mycoplasma*. Discrepancies with our results might be due to differences in sampling site and methodology applied. Also, information on biomass is lacking.

Strengths of this study are the untargeted approach to identify pathogen overgrowth and therefore potential bacterial and fungal co-infections in severely ill patients. Second, the ability to consider the complete microbial community, and relative abundance of individual taxa herein is a strength. Last, we presented a method incorporating the combination of information on biomass with relative overgrowth of specific (groups) of species, which provides additional useful information over other molecular detection methods^29^. Our study also has several limitations. Due to the general cross-sectional nature of our data, it was not designed to investigate microbiota changes over a longer period of time in relation to outcome. Another limitation is the lack of data on infection severity, immune-related treatments, and comorbidities. Also the small sample size of this pilot study provides low statistical power to execute further sub-analysis to understand the association between bacterial and fungal communities and outcome. In addition, our pilot study lacks a negative control cohort and was executed as a single-center study. For future studies it is therefore recommended to study respiratory microbiota on ICUs of multiple hospitals including patients with and without COVID-19 as well as patients in ICU admitted for other reasons than primary respiratory infection. Our methodology is also not suitable to provide information on antimicrobial resistance rather than can be extrapolated from the microbes identified. Finally, our methodology still requires significant time from sampling to results, which is similar to traditional culture methods^30^. Therefore, investment in further protocol adaptations is needed to reduce the turnaround time from sampling to results, allowing incorporation of these methods into clinical practice.

Nevertheless, our findings do suggest further studies on host-microbiome interactions, including, co-morbidities, exploring a relationship between the respiratory microbiota and severity, complications, and recovery of COVID-19 infections, are needed. Our data also support further studies on trying to understand whether these microbial changes in the respiratory communities facilitate SARS-Cov-2 infection or are a consequence of physiological and immunological processes underlying the clinical manifestations of COVID-19, and/or are the result of specific treatments, including antimicrobials. Longitudinal cohorts will help to discern the association between the (respiratory) microbiota and disease presentation and progression. Besides, a control cohort of ICU patients suffering from acute respiratory failure not caused by COVID-19 would be helpful to clarify whether the lung microbiota profiles we observed are generalizable across all (non-COVID-19) ICU-patient groups.

In summary, the methods presented could support clinical decision making regarding antimicrobial use as well as de-escalating treatment. Moreover, it may contribute to monitoring of (emerging) ventilator-associated infections, and identify potential overgrowth of pathogenic/inflammation-driven bacteria and fungi at an early stage of infection.

## Methods

### Study design and participants

In this proof-of-concept study, we investigated the bacterial and fungal community composition in lower respiratory tract secretions of 30 patients with proven COVID-19 infection admitted to the ICU of the Royal Infirmary of Edinburgh on or before the 2nd of April 2020. Most samples obtained were tracheal aspirates, with three additional sputum and one bronchoalveolar lavage sample. Specimens were collected according to standard clinical care guidelines. Respiratory samples were immediately transported to the diagnostic laboratory^31^, where SARS-CoV-2 particles were inactivated and nucleic acid stabilized by adding 3 ml of a 50:50 mixture of NucliSENS Lysis Buffer (bioMérieux, Marcy-l’Étoile, France) and RemelMicrotest Viral Transport Medium (Thermo Fisher, San Diego, CA, USA) to 1 ml of sample. Inactivated samples were stored at − 20 °C prior to transfer to the research laboratory for microbiome analyses. Demographic and clinical data were collected from electronic patient records.

Handling and testing of specimens and data for the study was carried out in accordance with the local ethical approval (South East Scotland SAHSC Human Annotated BioResource reference No.10/S1402/33). Provision and use of material was covered by Lothian NRS BioResource RTB approval (Ref: 15/ES/0094). All participants (or their guardians) signed an informed consent. All methods were performed in accordance with relevant guidelines and regulations.

### Procedures

Microbial DNA was isolated and processed as previously described^32^. In short, for bacterial community profiling, the V4 hypervariable region of the 16S-rRNA gene was amplified using barcoded primer pair 533F/806R^32^. To profile the fungal community, we targeted the ITS1 region using a two-step protocol as described by Illumina (Fungal sequencing and classification with the ITS Metagenomics Protocol) with the following modifications. The first amplification was performed using universal primers ITS1F/ITS2^33^ under the following conditions: the amplification mix contained 0.2 µL of Phusion^®^ Hot Start II High-Fidelity DNA Polymerase, 4 µL of 5 × Phusion^®^ Hot Start II High-Fidelity buffer, 0.4 µL dNTP (100 mM), 0.5 µL of each primer (10 µM), 0.6 µL of DMSO, 0.2 µL of BSA (10 mg/mL), 5 µL of the extracted DNA and 9.6 µL of DNA-free water. An initial denaturation step of 98 °C for 1 min was followed by 25 cycles of denaturation at 98 °C for 15 s, annealing at 55 °C for 45 s and extension at 72 °C for 45 s. Two subsequent limited-cycle amplification steps were performed to add Illumina adapters and dual-index barcodes. Five microliters of the “previous” PCR product served as template for the subsequent PCR performed under the same conditions as described above, but for 8 cycles and using PCR primers designed to integrate the sequence of 16S-rRNA gene primers (533F/806R) and barcoded primer pair 533F/806R that integrate Illumina multiplexing sequencing primers and adaptors for the second and third PCR respectively.

Amplicons were quantified by PicoGreen (Quant-iT™ PicoGreen^®^ dsDNA Assay Kit, Thermo Fisher) and pooled in equimolar amounts. Amplicon pools of samples and controls were sequenced using the Illumina MiSeq platform (San Diego, CA, USA) in two runs, containing 102 libraries and 43 samples in total. One sample was excluded from the final dataset because it was a clinical duplicate.

### Bioinformatic processing

Bioinformatic processing of 16S-rRNA gene reads was performed as previously described^34^ and included quality filtering/trimming, error correction, read assembly and binning reads in OTUs of 97% similarity. OTUs were annotated using the Silva database (version 119)^35^. We refer to OTUs using maximum genus-level annotations, combined with a rank number based on the abundance of each given OTU.

Fungal ITS1 amplicons were processed similarly to bacterial sequencing data, except a minimum merge length of 150 bp was applied to account for varying ITS1 sequence lengths. Taxonomic assignment of ITS1 OTUs against the UNITE QIIME release database version 01.12.2017 was performed using the RDP classifier in QIIME version 1.9^36^.

### Quality control

To control for contaminating DNA we included both DNA isolation and PCR controls. Following, we ran the *decontam* R-package^37^ for 16S-rRNA- and ITS-based data separately. Abundance and prevalence methods within the *decontam* R-package were applied to identify and exclude potential contaminant OTUs. As a positive control for the bacterial dataset we included a mock community previously described^34^ and for the fungal dataset a mock community containing *Penicillium spp., Aspergillus fumigatus, Aspergillus nidulans, Trichophytuminterdigitale, Candida albicans, Fusarium solani, Saccharomyces cerevisiae, Mucor spp.,* and *Exophiala dermatitidis.* Bacterial species were grown in BHI 37 °C overnight at 500 r.p.m. Fungal species were grown in Sabouraud liquid medium (Sabouraud Dextrose Broth, Oxoid, ThermoFisher) at 37 °C during 48 h at 500 r.p.m. 200 µL of pure culture of both, bacterial and fungal species, were used for DNA extraction. DNA concentration was quantified using PicoGreen and equimolar pooled except for *C. albicans* that was 10X less concentrated.

### Quantifying biomass

We quantified the DNA concentration of post-ITS1 PCR product by PicoGreen. For bacteria, we used qPCR to quantify the bacterial density^32^. We observed a significant linear relationship between the log-transformed 16S-rRNA qPCR data and the DNA concentration of post-V4 16S rRNA gene PCR quantified by PicoGreen (Supplementary Fig. 1).

### Statistical/data analysis

All analyses were performed in R version 3.6.3 within R studio version 1.2.5033 (Boston, MA). primarily with the packages vegan, phyloseq^38^, microbiome^39^, and ggplot2^40^. Benjamini-Hochberg (BH) adjusted P values (q values) were generated where appropriate. A *p* value and a *q* value of 0.05 were considered significant, unless otherwise stated. Patients characteristics were explored and represented by n (%) for categorical data and mean (standard deviation SD) or median (range, where range is expressed as minimum value—maximum value) for continuous data where appropriate (normally distributed or non-normally distributed according to Shapiro–wilk test, respectively). For both bacterial and fungal datasets first the relative abundances were calculated first by dividing the sequencing reads assigned to different taxa by the total number of reads per sample. Following, we calculated absolute abundances of bacteria and fungi per sample by multiplying the relative abundance of each taxon by the bacterial and fungal biomass, respectively. All patient samples were subjected to a similarity-based, unsupervised hierarchical clustering approach based on absolute abundances. To assess the optimal number of clusters we used a combination of the Caliński-Harabasz measure and Silhouette index. Clusters consisting of single samples were grouped in “Other”. Correlations between bacterial and fungal biomass were studied using linear models, and associations between bacterial and fungal clusters was determined by Chi Squared tests. Co-occurrence of the most abundant bacterial and fungal taxa (top 20 most abundant taxa based on mean absolute abundance data) were assessed by Spearman rank correlation.

## Supplementary Information

Supplementary Information.

## Data Availability

Sequence data that support the findings of this study have been deposited in the NCBI Sequence Read Archive (SRA) database with BioProject ID PRJNA740038.
