# Supplementary material for: Bacterial and fungal communities in tracheal aspirates of intubated COVID-19 patients: a pilot study
Source: Sci Rep. 2022 Jun 14;12:9896. doi: 10.1038/s41598-022-13482-w (PMC9196859; doi:10.1038/s41598-022-13482-w)
Supplement: Supplementary file 1 — Supplementary Information. [file 41598_2022_13482_MOESM1_ESM.pdf]

Supplementary figure1

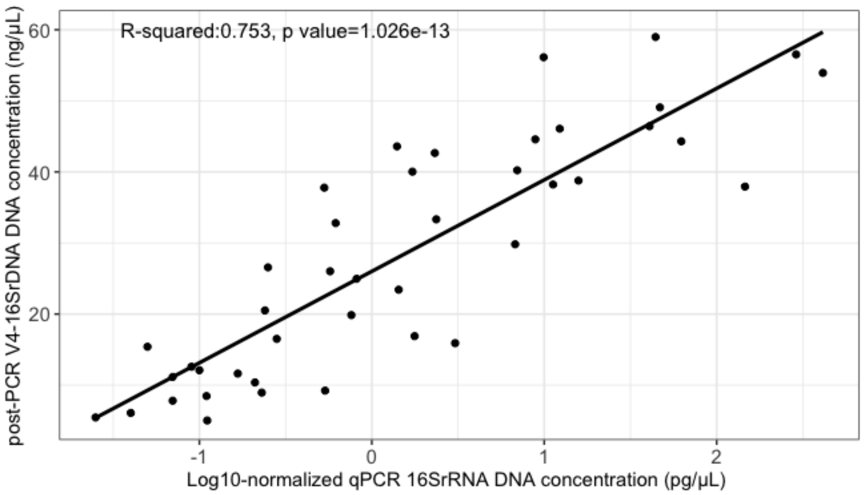

Supplementary figure2

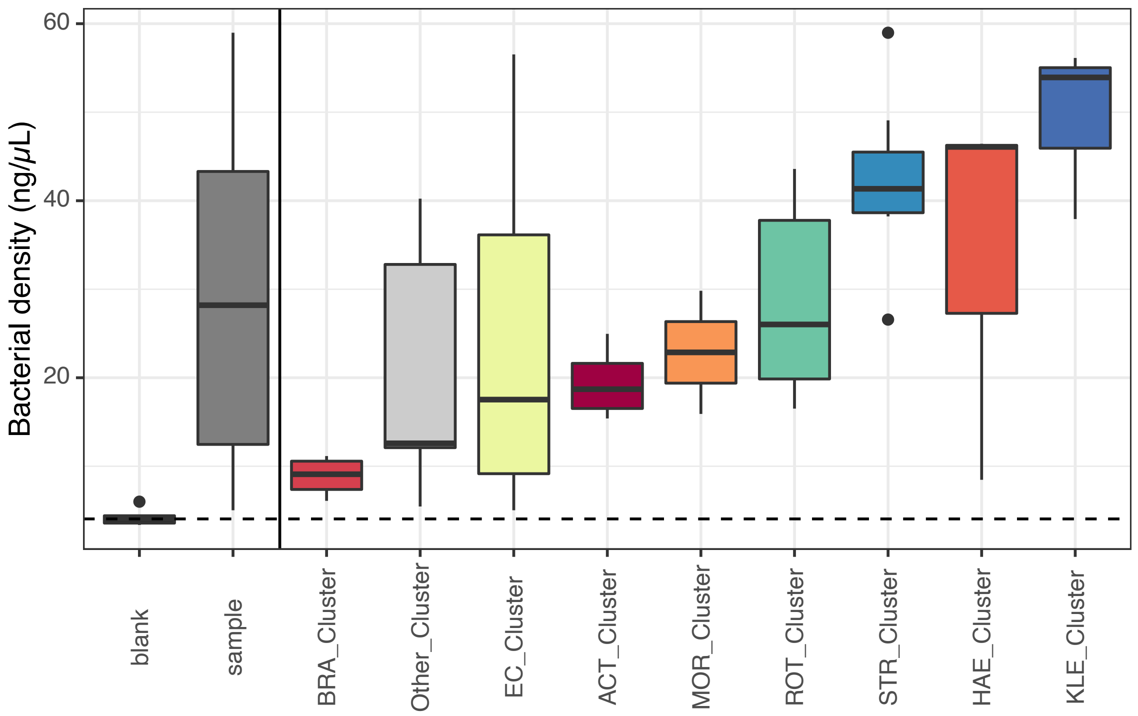

Supplementary figure 3

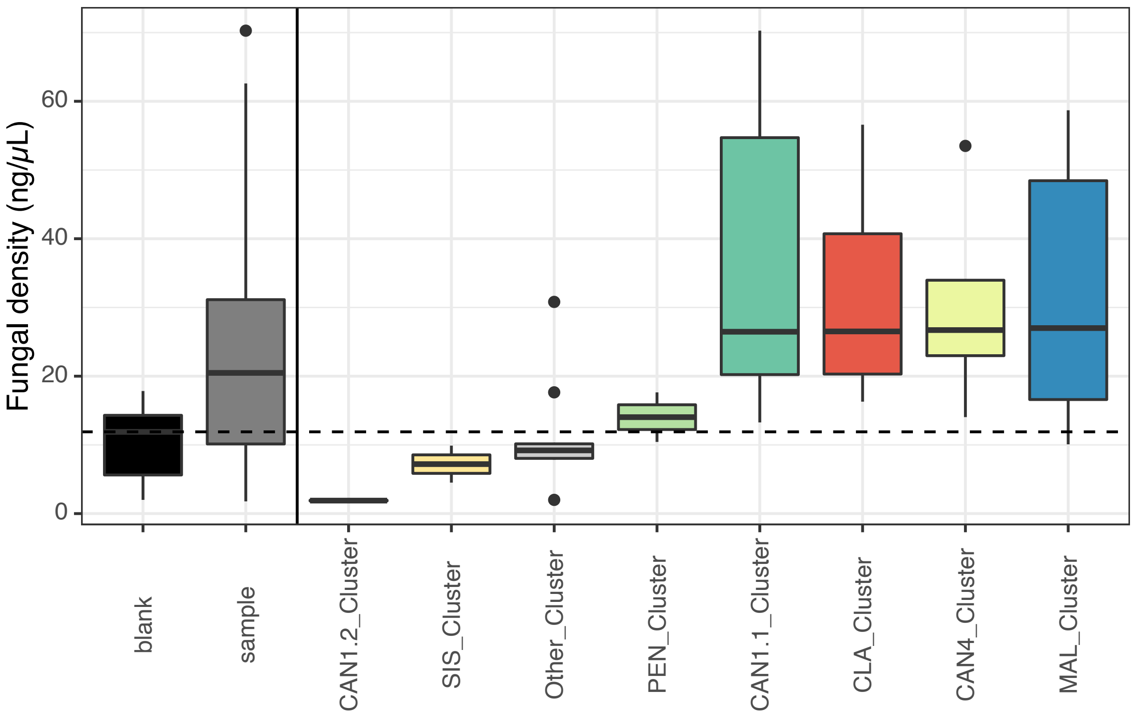

Supplementary figure 4

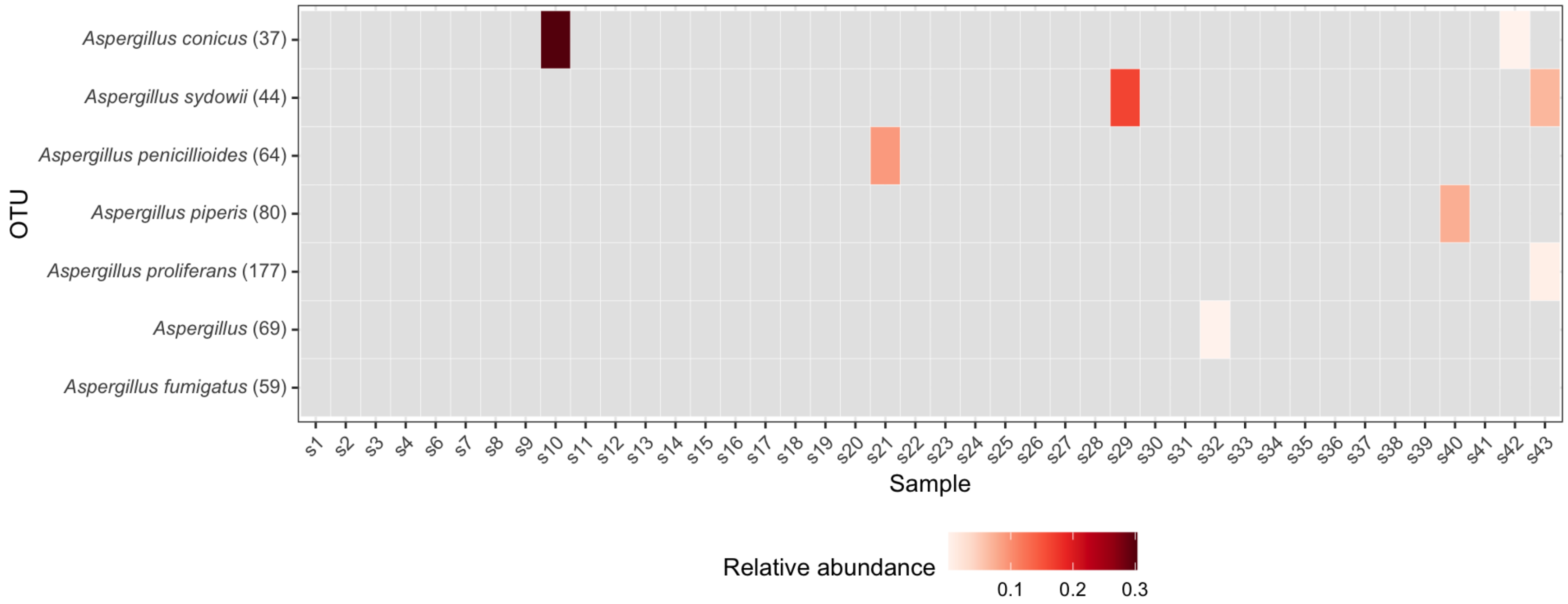

Supplementary figure 5

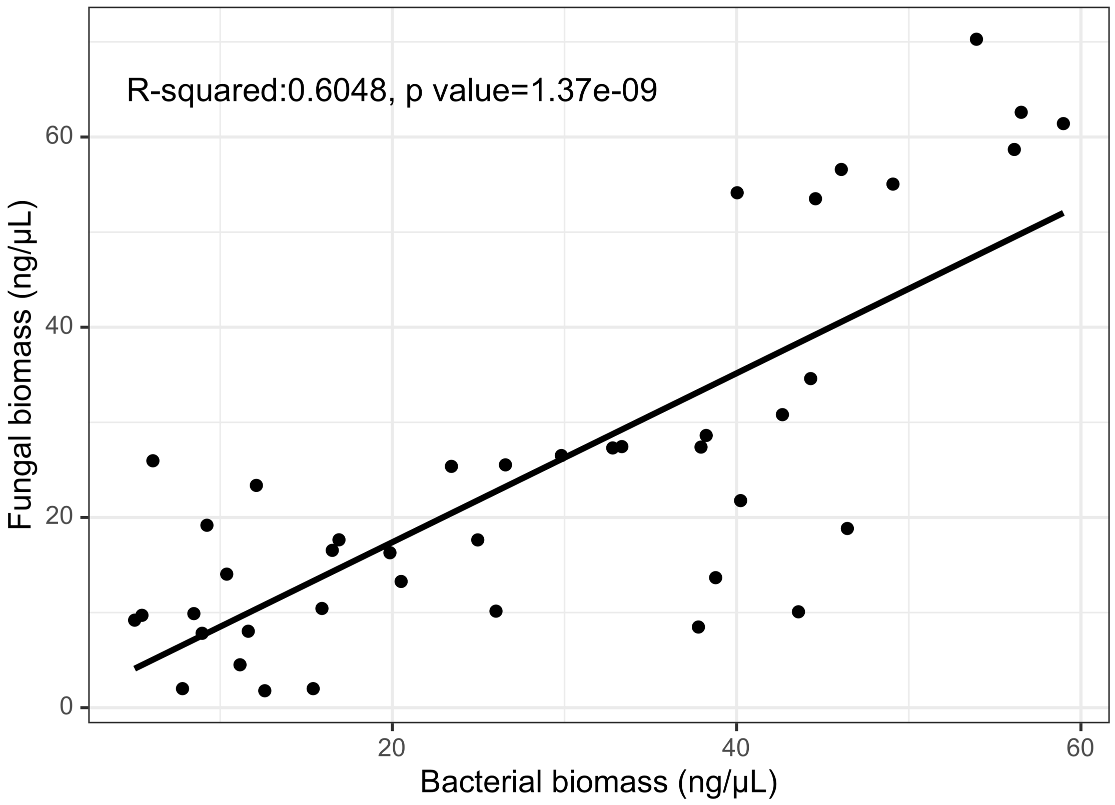

Supplementary figure 6

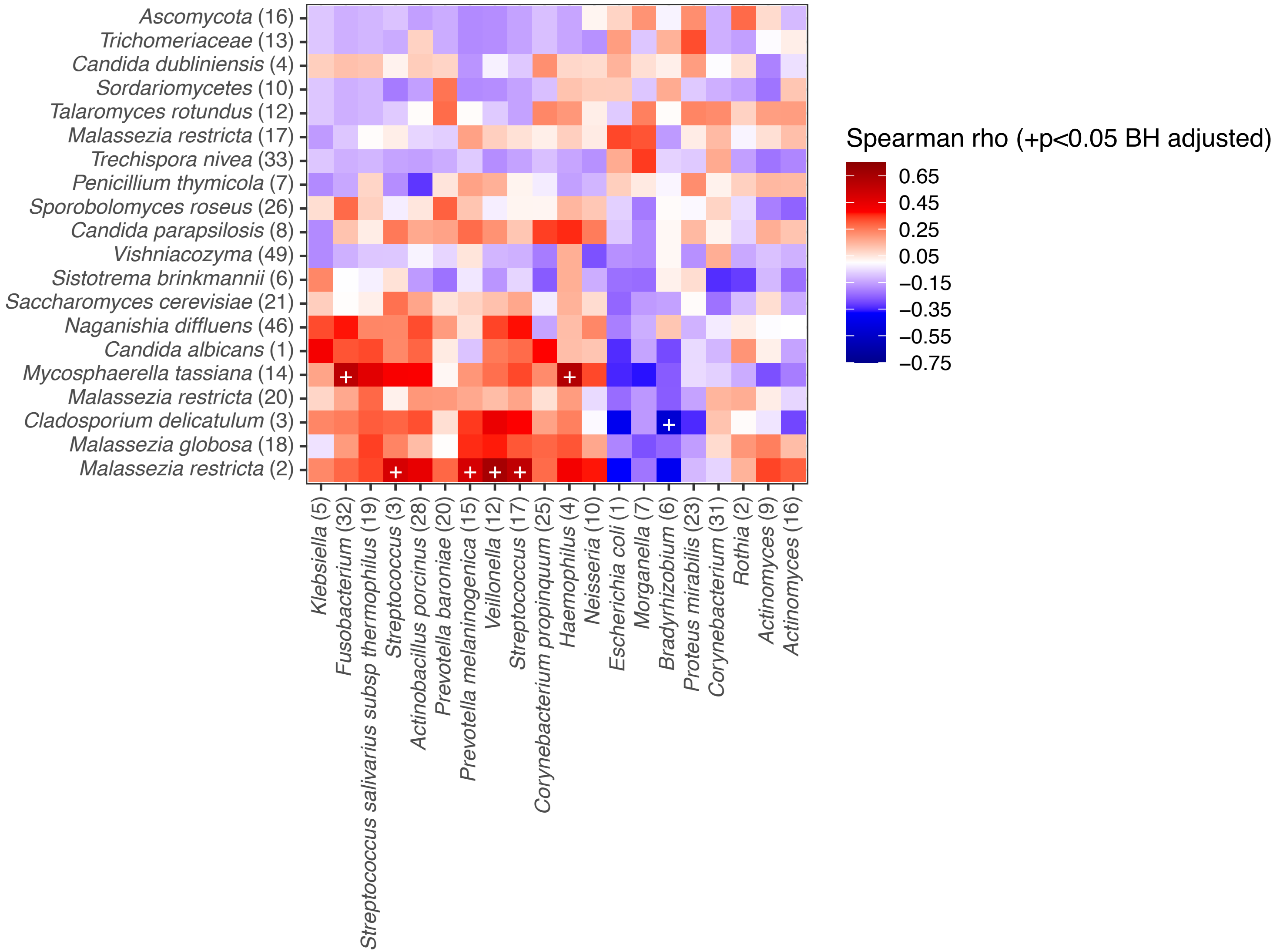

Supplementary figure 1. Linear relationship between log-10 normalized qPCR 16S rRNA DNA concentration in pg/µL and post-PCR V4-16S rRNA DNA concentration in ng/µL. Black line shows a linear model fit. R2 and *p* value are displayed.

Supplementary figure 2. Differences in bacterial biomass between clusters. Bacterial biomass is measured as post-PCR V4 16S-rRNA gene DNA concentration in ng/µL. Left panel includes boxplots showing the bacterial biomass in blanks and samples. Right panel includes boxplots showing the bacterial biomass in samples stratified by clusters. Clusters are ordered according to their median bacterial biomass. Dashed line indicates the median bacterial biomass in the blanks.

Supplementary figure 3. Differences in fungal biomass between clusters. Fungal biomass is measured as post-PCR ITS1 gene DNA concentration in ng/µL. Left panel includes boxplots showing the fungal biomass in blanks and samples. Right panel includes boxplots showing the fungal biomass in samples stratified by clusters. Clusters are ordered according to their median fungal biomass. Dashed line indicates the median fungal biomass in the blanks.

Supplementary figure 4. Heatmap showing the absolute abundance of OTUs classified under *Aspergillus* genus.

Supplementary figure 5. Relationship between bacterial and fungal biomass. Bacterial biomass is measured as post-PCR V4 16S-rRNA gene DNA concentration in ng/uL. Fungal biomass is measured as post-PCR ITS1 gene DNA concentration in ng/uL. Black line shows a linear model fit. R2 and *p* value are displayed.

Supplementary figure 6. Interdomain co-occurrence heatmap. Heatmap to show Spearman's correlation between absolute abundance of the 20 highest-ranked bacterial and fungal OTUs. White cross indicates statistically significant correlation at the level of (p adjusted < 0.05).
